# The Impact and Management of Gynaecomastia in Klinefelter Syndrome

**DOI:** 10.3389/frph.2021.629673

**Published:** 2021-02-12

**Authors:** Amr Abdel Raheem, Ahmed Said Zaghloul, Ahmed M. G. Sadek, Bilal Rayes, Tarek M. Abdel-Raheem

**Affiliations:** ^1^Andrology Department Cairo University Hospital, Beni Suef, Egypt; ^2^Faculty of Population Health Sciences, Institute for Women's Health, University College London, London, United Kingdom; ^3^King's College London, London, United Kingdom; ^4^Prosperite Occupational Health, Leicester, United Kingdom

**Keywords:** gynecomastia, Klinefelter syndrome, surgical management, non-surgical management, hypogonadism

## Abstract

Gynecomastia is defined as a palpable enlargement of the male breast, secondary to an increase in the glandular and stromal breast tissue. Gynecomastia is encountered in up to 80% of Klinefelter syndrome cases. The pathophysiology involves testosterone/estrogen imbalance. This review article will further explore the pathophysiology of gynecomastia along with the different lines of management.

## Background

Gynaecomastia can be defined as benign proliferation of the glandular tissue of the male breast which manifests clinically as breast enlargement with a palpable firm rubbery disc extending concentrically from the nipples (1, 2). It is to be differentiated from lipomastia which refers to breast enlargement due to accumulation of fatty tissue (3).

The aim of this mini-review article is to further explore the pathophysiology of gynecomastia along with the different lines of management, with emphasis on the Klinefelter patient population.

An open-label literature search was conducted using Medline from Pubmed. Generally, preference was given to the latest/most cited publications authored in English.

## Prevalence of Gynecomastia

Gynaecomastia is by far the most common condition affecting the male breast. It has been estimated that 30–70% of males will experience gynaecomastia at some stage in their life. Statistics from the USA show that 36% of adult young men and 57% of older men will have this condition in any of its different grades of severity (4). It can therefore be deduced that it can be as common as one in every three adult men and one in every two elderly men (4).

Thirty to sixty percentage of boys will experience pubertal gynaecomastia but this is usually transient and resolves spontaneously whereby only 8% will have persistent pubertal gynaecomastia after 3 years (5). Thirty to 50% of the cases present as bilateral gynaecomastia (i.e., it can also present unilaterally) (6). In Klinefelter syndrome (KF) gynecomastia is seen in about 80% of cases (7).

## Pathophysiology of Gynecomastia in Klinefelter's Syndrome

In essence, the pathophysiological process of gynecomastia involves an imbalance between free estrogen and free androgen actions in the breast tissue causing a low T:E2 ratio. This can happen in a number of physiological and pathological conditions such as puberty, obesity, primary hypogonadism, secondary hypogonadism due to hypothalamo-pituitary disorders, liver cirrhosis, renal failure, estrogen producing tumors, medication which has an antiandrogenic or estrogenic effect (8–10).

KF is associated with primary testicular failure and hypogonadism with low or low normal testosterone concentrations (10–12). KF is also associated with an increase in the activity of aromatase enzyme which will cause an increase in the peripheral conversion of testosterone to oestradiol (13).

## Negative Effects of Gynecomastia on Patients With Klinefelter's Syndrome

Gynecomastia can cause significant distress, negative body-image issues and embarrassment for the affected individuals. It could even prevent them from normal social interaction, engaging with a partner, participating in sports and other activities (this could be due to the feeling of shame, embarrassment and fear of rejection or being made fun of) (14, 15).

KF is also the only cause of gynecomastia that clearly carries an increased risk of breast cancer 10–20-fold greater than normal. The cancer risk could be related to the extra X-chromosome (7). This warrants the treatment of gynecomastia (see Table 1) in KF patients as a necessity rather than purely for aesthetic/image-related purposes.

**Table 1:** Treatment lines of gynecomastia.

**Treatment of Gynecomastia**	
**Non-surgical**	**Surgical**
*- Antioestrogens* *- Aromatase enzyme inhibitors* *- Testosterone replacement therapy*	*Conventional liposuction* *- Ultrasound assisted liposuction* *- Power assisted liposuction* *- Liposuction using radiofrequency energy* *- Laser assisted lipolysis* *- Mastectomy*

## Diagnosis and Evaluation of Gynecomastia in Men With KF

The diagnosis of gynecomastia is made clinically by the presence of subareolar firm glandular tissue ≥ 2 cm in diameter. Differential diagnosis includes lipomastia and breast cancer. Lipomastia involves the accumulation of soft fatty tissue, while clinical signs of breast cancer include the palpation of a hard, asymmetric and eccentrically located lump and possible skin and nipple changes. If cancer is suspected mammography, ultrasonography and biopsy are indicated (1, 3, 4).

## Treatment of Gynecomastia

### Non-surgical Treatment of Gynecomastia

Medical treatment is likely to be beneficial if implemented during the early proliferative phase. After gynecomastia has been present for more than 1 year, the glandular structure is replaced by stromal hyalinization and fibrosis. As a result, breast tissue is less likely to respond to medication (16).

#### Antioestrogens (Selective Estrogenic Receptor Modulators (SERMs), Tamoxifen and Raloxifene)

Compete with estrogen at receptor sites in the breast tissue and inhibit transcription of growth genes. This appears to be effective in patients with short term development of gynecomastia (17–19).

### Aromatase Enzyme Inhibitors Such as Anastrozole

Will prevent the peripheral conversion of testosterone into oestradiol and has been used in men with hypogonadism and gynecomastia (9).

### Testosterone Replacement Therapy

Leads to the resolution of gynecomastia in many hypogonadal men. However, because it can be aromatised to oestradiol, testosterone therapy may worsen gynecomastia and can even lead to new onset of gynecomastia in some cases. For this reason, non-aromatisable androgens like dihydrotestosterone (DHT) are used (9). Testosterone therapy can also be combined with an aromatase enzyme inhibitor (20).

### Surgical Treatment of Gynecomastia

Generally, the objectives of surgical treatment include, but are not limited to: flattening of the thoracic region; removal of redundant skin; symmetrisation between the two sides; containment of scars (16).

### Conventional Liposuction

Liposuction is the surgical removal of subcutaneous fat by means of aspiration cannula introduced through small skin incisions, assisted by suction. Synonyms used in literature include liposuction surgery, suction-assisted lipectomy, suction lipoplasty, fat suction, blunt suction lipectomy, and liposculpture (21, 22). Male breasts are one of four most requested areas for liposuction in men (after love handles, abdomen, and submental fat). The main reason is the cosmetic inconvenience and the loss of self-confidence produced by a feminine self-image (23).

### Ultrasound Assisted Liposuction

The fat is more selectively emulsified, leaving the higher-density fibroconnective tissues relatively undamaged. This technique effectively removes dense adipose tissue within the fibrous parenchymal framework of the male breasts, and this has proved especially effective in the treatment of gynecomastia (24).

### Power Assisted Liposuction

This is gaining popularity in liposuction procedures. The oscillating movement of the cannula easily mimics, in a strain-less and more controlled fashion, the work of a surgeon during traditional suction-assisted lipoplasty. The vibration allows easy penetration of even fibrous fat, while generating no thermal energy, thus no risk of cutaneous burns as compared with the ultrasound-assisted technique (25).

### Liposuction Using Radiofrequency Energy

Using a blunt-nose suction cannula and simultaneously aspirating the coagulated tissue, this procedure results in coagulation of adipose, vascular and fibrous tissue (26).

### Laser Assisted Lipolysis

This has the advantages such as: excellent patient tolerance, quick recovery time, and the additional benefit of dermal tightening (27).

### Mastectomy

Various incisions and techniques have been described for the subcutaneous mastectomy. The most commonly used ones are Webster's intra-areolar, peri-areolar, and circum-areolar incisions (28).

The use of the endoscope has been described for the treatment of fibrous gynecomastia. This technique has improved the final outcome with less scarring, short operative time, minimal complications and good aesthetic results (29). The amplification provided by the endoscope also makes the whole operating field clear on the monitor screen, thus providing a wider field (30).

## Conclusion

Gynaecomastia is highly prevalent issue for males of all ages. There are various options for treatment with the surgical approach being the most efficacious for adequate long-term treatment. The increased risk of breast cancer and the negative body image and distress it causes has rendered the treatment of gynecomastia in the KF patient population a matter of necessity rather than purely for aesthetic/image-related purposes.

## Author Contributions

AR was involved in ideation as well as writing and revision of the final article. AZ and AS were tasked with the collection of materials. BR was also involved in the collection of scientific material, language revision, and submission of the article. TA-R was responsible for writing the body of the article. All authors contributed to the article and approved the submitted version.

## Conflict of Interest

The authors declare that the research was conducted in the absence of any commercial or financial relationships that could be construed as a potential conflict of interest.
